# Scaling and Benchmarking an Evolutionary Algorithm for Constructing Biophysical Neuronal Models

**DOI:** 10.3389/fninf.2022.882552

**Published:** 2022-06-17

**Authors:** Alexander Ladd, Kyung Geun Kim, Jan Balewski, Kristofer Bouchard, Roy Ben-Shalom

**Affiliations:** ^1^Electrical Engineering and Computer Sciences, University of California, Berkeley, Berkeley, CA, United States; ^2^NERSC, Lawrence Berkeley National Laboratory, Berkeley, CA, United States; ^3^Helen Wills Neuroscience Institute & Redwood Center for Theoretical Neuroscience, University of California, Berkeley, Berkeley, CA, United States; ^4^Scientific Data Division and Biological Systems and Engineering Division, Lawrence Berkeley National Laboratory, Berkeley, CA, United States; ^5^Neurology Department, MIND Institute, University of California, Davis, Sacramento, CA, United States

**Keywords:** biophysical neuron model, high performance computing, evolutionary algorithms, non-convex optimization, strong scaling, weak scaling, electrophysiology

## Abstract

Single neuron models are fundamental for computational modeling of the brain's neuronal networks, and understanding how ion channel dynamics mediate neural function. A challenge in defining such models is determining biophysically realistic channel distributions. Here, we present an efficient, highly parallel evolutionary algorithm for developing such models, named *NeuroGPU-EA*. *NeuroGPU-EA* uses CPUs and GPUs concurrently to simulate and evaluate neuron membrane potentials with respect to multiple stimuli. We demonstrate a logarithmic cost for scaling the stimuli used in the fitting procedure. *NeuroGPU-EA* outperforms the typically used CPU based evolutionary algorithm by a factor of 10 on a series of scaling benchmarks. We report observed performance bottlenecks and propose mitigation strategies. Finally, we also discuss the potential of this method for efficient simulation and evaluation of electrophysiological waveforms.

## 1. Introduction

Since Hodgkin and Huxley's seminal work on recording and mathematically formulating the activity of the giant squid axon (Hodgkin and Huxley, 1952), great progress has been made in understanding the electrical properties of single neuron models. The development of the patch-clamp technique (Sakmann and Neher, 1984), which enabled recording neurons in finer resolution, and the work by Rall (1959, 1962, 1964) on modeling the cable properties of dendritic trees have been the basis of extensive research in numerical methods for compartmental neuron models (Rall, 2009). The formulation of electrical properties of neurons in digital computers (Hines, 1984; Carnevale and Hines, 2006; Bower and Beeman, 2012) enabled simulating experimental observation in computational models (Traub et al., 1991, 2005; De Schutter and Bower, 1994; Mainen et al., 1995). These advancements have brought the field of computational neuroscience closer to realistically modeling biological neurons on computers (Markram et al., 2015; Nogaret et al., 2016; Ben-Shalom et al., 2017; Bouchard et al., 2018; Gouwens et al., 2018; Daou and Margoliash, 2020; Spratt et al., 2021). Multi-compartmental biophysical models, such as the Mainen & Sejnowski model (Mainen and Sejnowski, 1996) and those comprising the large-scale neocortical column simulation (Markram et al., 2015; Ramaswamy et al., 2015; Gouwens et al., 2018; Billeh et al., 2020), aim to simulate the electrical properties of single neuron. The NEURON (Hines et al., 2004; Carnevale and Hines, 2006) simulation environment is a commonly used software for simulating how different channel conductances contribute to the electrical activity of the neuron. However, constraining the conductance of various membrane ion channels and biophysical properties of the membrane remains a major obstacle in fitting these models to experimental data (Prinz et al., 2004; Druckmann et al., 2007; Almog and Korngreen, 2016; Nogaret et al., 2016). As the number of free parameters that characterize the neuronal model increase, so does the cardinality of the optimization search space, thus making the optimization less tractable. Adding more parameters that govern channel and membrane dynamics makes the simulated neuron more specific to a physical neuron, but also adds more unknown variables with complex relationships. Thus, there exist trade-offs between the amount of detail in the model, computation time, computational power, and the questions that need to be answered by such models (Eliasmith and Trujillo, 2014; Almog and Korngreen, 2016; Sáray et al., 2020). Researchers must make limiting assumptions to constrain the number of free parameters to maintain feasible simulation and model fitting times.

With increasing model complexity comes the need for more efficient optimization methods. One challenge with constraining the parameters of electrophysiological neuron models is that the search space of possible model parameters is large. Furthermore, neurons with substantially different parameters can produce qualitatively similar responses (Goldman et al., 2001; Golowasch et al., 2002; Prinz et al., 2003). However, a small perturbation in the conductance of a single channel parameter can have a significant impact on the simulated voltage trace. In constraining single neuron parameters, there are several approaches including brute force search, Monte Carlo optimization algorithms such as evolutionary algorithms and simulated annealing, or heuristic algorithms (Keren et al., 2005; Druckmann et al., 2007; Van Geit et al., 2007, 2008, 2016). For the construction of biophysical neuron models in this paper, we chose to use the evolutionary algorithm (EA), a prevalent method for this optimization problem (Vanier and Bower, 1999; Keren et al., 2005; Druckmann et al., 2011; Masoli et al., 2017; Gouwens et al., 2018; Ben-Shalom et al., 2020). Our objective function is constructed from score functions comparing electrophysiological firing properties between simulated and experimental target voltages (Druckmann et al., 2007). This multi-objective optimization (MOO) is formulated using the Indicator-Based evolutionary algorithm (IBEA) (Zitzler and Künzli, 2004). EA searches for solutions that present optimal trade-offs between electrophysiological score functions. We focused on efficiently scaling EA to mitigate computational bottlenecks and highlight potential benefits. We considered the construction of the objective function outside the scope of this work. We show the motivation for accelerating this algorithm through scaling the parameter search algorithm on a motivating example model.

Advancements in chip capacity (Schaller, 1997) and software for high performance computing (HPC) platforms (Fan et al., 2004; Strohmaier et al., 2015) have the potential to accelerate electrophysiological simulation (Bouchard et al., 2018) and consequently the EA algorithm. We focused on benchmarking two classes of software modules—neuron simulators and electrophysiological spike train feature extractors, due to their central importance in EA. While it is important to experiment with performance benchmarks that are specific to individual modules it is also important to develop benchmarks that assess the application of combinations of modules. This study draws from previous work in benchmarking for computer science (Hoefler and Belli, 2015; Bouchard et al., 2016; Coleman et al., 2019; Wu et al., 2019) by applying established performance benchmarks to software for neuron simulation and biophysical modeling. These experiments utilize two well-established benchmarking strategies: strong scaling and weak scaling (Bailey et al., 2010; Balasubramanian et al., 2020). In total, we used three benchmarks:

- “Compute Fixed and Problem Scales”: The number of neuron models used in EA increases across experiments but the allocation of computing nodes, cores, and/or GPUs available is fixed.
- “Compute Scales and Problem Fixed” or strong scaling: The allocation of computing nodes, cores, and/or GPUs increases across experiments but the number of neuron models used in EA is fixed.
- “Compute Scales and Problem Scales” or weak scaling: The allocation of computing nodes, cores, and/or GPUs and the number of neuron models used in EA both increase across experiments at a fixed ratio.

These experiments investigate the impact of modularizing EA using different software tools for simulation and electrophysiological feature extractors. Using this experimental design in conjunction with various software and hardware configurations demonstrates the state of the art, challenges, and opportunities, related to efficiently utilizing HPC resources for complex biophysical neuronal modeling.

Adapting well-known performance benchmarks to EA helps understand how the algorithm can scale using different configurations of computational resources and software modules. While (Knight and Nowotny, 2018; Van Albada et al., 2018; Criado et al., 2020; Kulkarni et al., 2021), all provide relevant examples of benchmarking simulation modules and computational platforms, such as neuromorphic hardware, there is a gap in benchmarking the performance of such simulators applied to the neuron fitting problem. This work aims to address this gap by evaluating the run time performance of the evolutionary algorithm as a method to construct biophysical neuron models. Thus, the principal contributions of this paper are as follows:

1. We present an optimized implementation of the evolutionary algorithm, *NeuroGPU-EA*, that aims to accelerate the time it takes to fit a biophysical neuronal model by leveraging parallelism on high performance GPU nodes.
2. We benchmark the run time of this algorithm using well-established performance benchmarks *weak scaling* and *strong scaling*.
3. We vary implementation by:

3a. Running experiments on CPU only nodes with the *CPU-EA* algorithm or CPU-GPU experiments with *NeuroGPU-EA* algorithm.
3b. Using different electrophysiological feature extraction libraries.
3c. Using different GPU-based neuron simulation modules, such as CoreNeuron in *CoreNeuron-EA*.

In the following sections of this paper, we first give a brief overview of the implementation of the evolutionary algorithm and how simulation and feature extraction drive the algorithm toward increasingly realistic neuronal modeling. Next, we specify the hardware and software on the machines we used. Then we give a description of National Energy Research Scientific Computer Center's (NERSC) supercomputer Cori^1^, which was used to test the scaling of each variation of this algorithm. The experimental design allows for the comparison of different algorithms, using GPU and CPU, as well as different software modules in the simulate-evaluate loop. Subsequently, we demonstrate the results of such experiments and discuss the implications. We show how scaling the evolutionary algorithm for an example cell results in a more realistic model. Finally, we discuss challenges faced in benchmarking EA and future steps for analysis.

## 2. Methods

### 2.1. Evolutionary Algorithm

The optimization problem considered in this paper is the fitting of biophysically accurate parameters of a neuron using evolutionary algorithms (EAs). EAs are a class of optimization methods that rely on natural selection in a population through biologically inspired operators such as mutation, crossover, and fitness-based selection (Mitchell, 1998). This version of EA encodes solutions to an optimization problem into continuous vector representations of neuron model parameters. We refer to this group of parameterized neuron models as the “population” and a single model as an “individual”. EAs represent the quality of these vector representations by evaluating an objective function that takes this population as an input and compares the models' responses to experimental data. The algorithm is known as the (μ, λ) evolutionary algorithm (Beyer and Schwefel, 2002; Beyer, 2007) and is implemented using DEAP (Fortin et al., 2012) and BluePyOpt^2^ (Van Geit et al., 2016). In this implementation, μ and λ are the size of the parent population and the number of offspring to produce for the next generation, respectively. The parameter *cxRate* is the probability that an offspring was produced by crossover and the parameter *mutRate* is the probability that an offspring is produced *via* mutation^3^. Mutation is a perturbation of one or more parameters and crossover is a combination between a pair of parameter sets. The function VARIATION in the EA algorithm, Algorithm 1, applies mutation, reproduction, or crossover exclusively to each individual, or pair in the case of crossover, to produce λ new offspring from a μ sized parent generation.

**Algorithm 1:** Evolutionary Algorithm

1:	**procedure** OPTIMIZE(*μ*, λ, cxRate, mutRate nGenerations)
2:	parents ← INITIALIZE()
3:	hof ← []
4:	parents.scores ← EVALUATE(parents)
5:	**for** *generation* ← 1, *nGenerations* **do**
6:	offspring ← VARIATION(parents, λ, cxRate, mutRate)
7:	offspring.scores ← EVALUATE(offspring)
8:	population ← parents + offspring
9:	parents ← SELECT(population, *μ*) ⊳ keep *μ* individuals using indicator value tournament selection (Zitzler and Künzli, 2004)
10:	hof ← hof.*update*(population) ⊳ hof tracks 10 lowest scoring models
	**end**
11:	**return** argmin *sum*(hof_i_.scores)⊳ the best model has the hof_i_ ∈ hof
	lowest sum of scores

**Figure 1:**
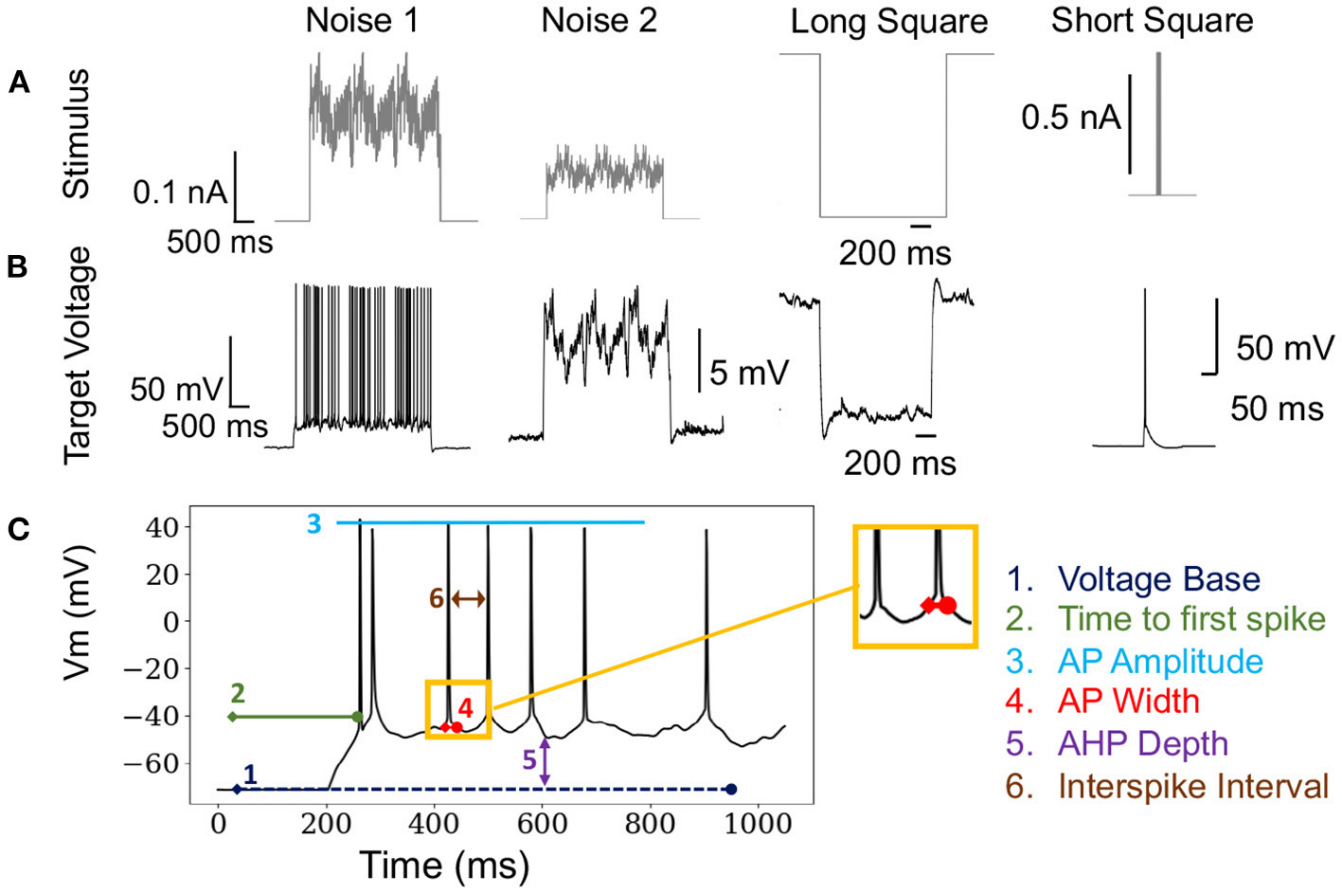
Stimuli and electrophysiological score functions used in algorithm: **(A)** Various stimuli used in the fitting procedure of EA. **(B)** Corresponding target voltages that are recorded from patch clamp experiments as a result of the stimuli in **(A)**. **(C)** Demonstrates how electrophysiological score functions are computed on a single trace. These score functions are used to compare target and simulated firing traces.

Formally, the optimization problem posed in this paper defines an individual *i* as $x_{i}\in {\mathbb{R}}^{13}$. Boldface ***x*** denotes a one-dimensional vector. The entire population is defined as *X*∈ℝ^13 × *N*^, where 13 is the number of free parameters of the neuron model and *N* is the size of the population (typically 50–5,000). The OBJECTIVE FUNCTION is computed using electrophysiological score functions, thus the term “score function” refers to one electrophysiological feature and the term objective function refers to the function characterizing the joint optimization such score functions (MOO). Initially, a model ***x*_*i*_** is simulated using *s*∈*S* stimuli, shown in Figure 1A, and evaluated against an experimental waveform, shown in Figure 1B, using *F* electrophysiological score functions, shown in Figure 1C. This procedure results in a set of scores for each individual. These scores are computed across each stimuli and score function (Druckmann et al., 2007). Then, BluePyOpt (Van Geit et al., 2016) uses an indicator based objective function (IBEA) that computes binary comparisons between individuals and their respective electrophysiological scores. These comparisons are calculated using the sum of indicator functions of the form *I*({***x***_*i*_}, {***x***_*j*_}), resulting in an indicator based fitness value, as referenced in line 6 of Algorithm 1. This definition of fitness is derived from Zitzler and Künzli (2004). The aforementioned μ individuals are obtained through an iterative process that acquires the winner of a tournament of binary comparisons between all individuals until μ individuals have been selected. The selected μ individuals, termed the “parents”, are used to produce a new set of offspring for the subsequent generation, as demonstrated in line 4 of Algorithm 1. After all individuals in the population, consisting of offspring and parents, are scored, the 10 individuals with the lowest sum of score functions are added to a hall of fame. The hall of fame has no impact on the evolution of the population, as it tracks the 10 lowest scoring individuals over all generations of EA. When the EA algorithm has terminated, on line 11 Algorithm 1, the lowest scoring individual is selected from the hall of fame.

In total, we used a set 18 stimulations consisting of 8 long square, 6 noisy, 2 short square, and 2 ramp stimuli, as represented in Figure 1A. In benchmarking tasks 3.2,3.3, 3.4 and 3.5, stimuli were chosen in a random order. The optimization in Section 3.6 used the same stimuli as benchmarking tasks 3.2,3.3, 3.4. However, the optimization in Section 3.7 only utilized the 8 long square stimuli. We chose to benchmark a diverse set of stimuli as the practice of EA for fitting neuron model parameters utilizes a wide range of stimuli, including passive stimuli not represented in this study. The full list of score functions is included as a Supplementary Material.

### 2.2. Implementations

In our implementation of EA, we used 20 scoring electrophysiological score functions from Blue Brain Project's Electrophys Feature Extraction Library (eFEL) library^4^ (Van Geit et al., 2016). The total offspring score is the unweighted sum of the selected score functions. Figure 1C is an illustration of how these scoring functions are computed on a single trace. The size of EA is defined as having cardinality *N* × *S* × *F*, which represents the population size × the number of stimuli presented × the number of score functions used. As mentioned above, the population for the evolutionary algorithm is comprised of parameter sets for the multi-compartment neuron model. We used a layer 5 thick tufted pyramidal neuron from the Blue Brain Project (Ramaswamy et al., 2015) with 13 different ionic channel parameters in the axon, soma, and dendrite. This cell morphology and parameterization can be found in Blue Brain Model portal^5^ as L5 TTPC1 cADpyr232 1. The Supplementary Table 1 shows how the parameters were distributed across axonal, somatic sections, as well as the upper and lower optimization bounds for each conductance. Supplementary Table 1 also shows that some of these parameters were modeled separately in the soma and the axon. The model used in Section 3.7, **Figure 7**, and Supplementary Figure 1 does not include a parameter for non-specific cation current *I*_*h*_ but the model used in the benchmarking Sections 3.2, 3.3, and 3.4 did include this parameter. This channel was not included in the optimization to reduce the complexity of the optimization task.

In the objective function Algorithm 2 there are three opportunities to implement parallelism:

1. **Population level parallelism**: run the simulate-evaluate loop in parallel across the entire population.
2. **Stimuli parallelism**: run all the simulations for each stimulus in parallel.
3. **Score function parallelism**: run all the score functions in parallel.

**Algorithm 2:** Objective Function

1:	**procedure** EVALUATE(offspring)
2:	scores ← {}
3:	**for all** *stim* ∈ *Stims* **do** ⊳ stimuli parallelism
4:	responses ← SIMULATE(offspring, stim)
5:	**for all** *scoreFunction* ∈ *scoreFunctions* **do** ⊳ score function parallelism
6:	scores[*scoreFunction*] ← *scoreFunction*(responses, target)
	**end**
	**end**
7:	**return** scores

**Figure 2:**
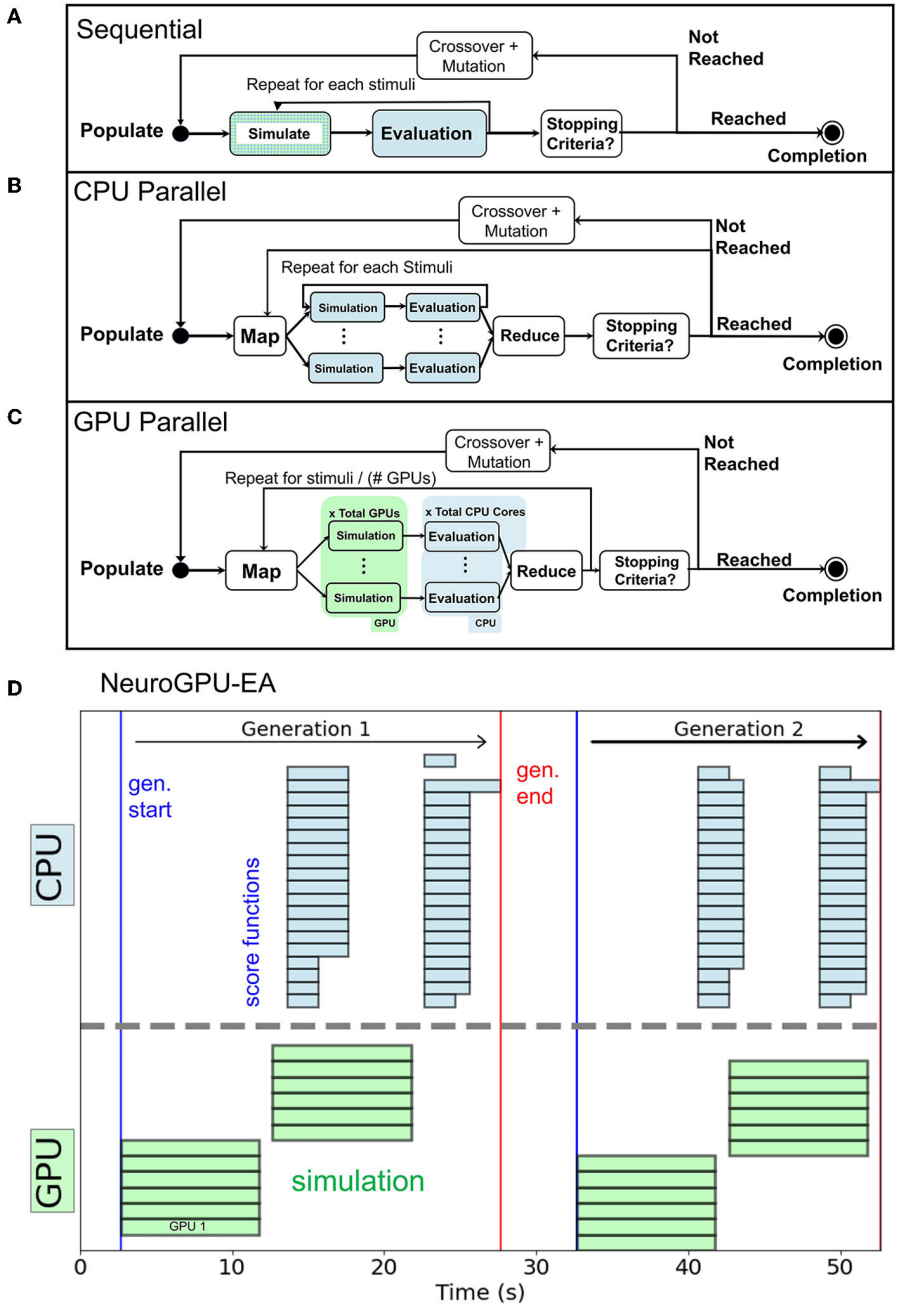
**(A)** Sequential execution EA. **(B)** *CPU-EA* maps the simulation/evaluation of a model to a single core. **(C)** *GPU-EA* maps each stimuli to a GPU, then scores the simulation in parallel on each CPU core. **(D)** Timeline of *NeuroGPU-EA* for two generations. The algorithm starts new stimuli on GPUs while the CPUs are still completing the previous ones.

In the objective function Algorithm 2, scores and responses are lists containing the voltages and scores for each individual of the population respectively. The objective function can be implemented as a triple for loop by including an initial loop over the population. Alternatively, Algorithm 2 implements a double for loop by defining scores as a vector of scores corresponding to each individual. Each stimulus response and score are computed without using information about other simulations, other electrophysiological score functions, or individuals in the population. Thus, the problem is embarrassingly parallel (Herlihy and Shavit, 2012). For reference, the sequential representation is summarized in Figure 2A. Our *CPU-EA* and *NeuroGPU-EA* algorithm took advantage of this feature in the following ways.

- *NeuroGPU-EA* employed all three approaches to implement parallelism, as demonstrated in Figure 2C. (i) The population level parallelism was achieved by dividing the entire population (MPI_SCATTER) across nodes and then aggregating (MPI_GATHER) at the rank 0 node at the end of evaluation. (ii) The simulations for each stimuli were computed in parallel across each available GPU. (iii) Each electrophysiological score function was computed in parallel on CPUs once the simulation responses were obtained.
- *CPU-EA* implementation, shown in Figure 2B, was parallelized over the population and one CPU core per individual was allocated using IPyParallel^6^. The parallelized *CPU-EA* procedure was run in parallel across the entire population (MAP) and aggregated (REDUCE) into a list once all scores have been calculated. Thus, *CPU-EA* leverages population level parallelism across all available CPU cores.

For *NeuroGPU-EA*, if there are more stimuli than GPUs available, it is necessary to launch batches of simulations while the CPU cores handle electrophysiological score function evaluation for the previous batch of stimuli. This case is demonstrated in Figure 2D and will be referenced in Section 3.5 in experiments that scale up the number stimuli used in EA. We compute scores on CPU and acquire additional CPU-GPU data transfer cost because we did not have access to a GPU implementation of the eFEL library.

### 2.3. Hardware

The experiments presented here were executed on the Cori-GPU cluster at NERSC^7^. Each Cori GPU node has two sockets of 20-core Intel Xeon Gold 6148 (“Skylake”) CPUs with 384 GB DDR4 RAM memory and a clock rate of 2.40 GHz. Each of these nodes also has 8 NVIDIA Tesla V100 (“Volta”) GPUs, connected with NVLink interconnect, each with 16 GB HBM2 memory. We used Cray's Programming Environment version 2.6.2. Allocated nodes were chosen by the batch system (SLURM 20.11.8) and were allocated exclusively to eliminate on-node interference. The system uses InfiniBand host network adapters (HCA) and network interface cards (NICs). Each Cori CPU node has two sockets, each socket is populated with a 2.3 GHz 16-core Haswell Intel Xeon Processor E52698 v3. Each core supports 2 hyperthreads, and has two 256-bit-wide vector units 36.8 Gflops/core (theoretical peak), 1.2 TFlops/node (theoretical peak) and 2.81 PFlops total (theoretical peak). Each node has 128 GB DDR4 2133 MHz memory (four 16 GB DIMMs per socket) and 298.5 TB total aggregate memory. The interconnect is Cray Aries with Dragonfly topology with 45 TB/s global peak bisection bandwidth. We used Cray's Programming Environment version 2.6.2. Allocated nodes were chosen by the batch system (SLURM 20.11.8) and were allocated exclusively to eliminate on-node interference. For all experiments, we used Cori SCRATCH which is a Lustre file system designed for high performance temporary storage of large files. All experiments were run on x86 64 computing architecture, SUSE Linux Enterprise 15 and kernel 4.12.14-150.75-default.

### 2.4. Software

We used GCC compiler version 8.3.0, CUDA version 11.1.1, OpenMPI version 4.0.3, Python 3.8.6. As in previous work (Ben-Shalom et al., 2012, 2013, 2022), the evolutionary algorithm was implemented using the DEAP 1.3 (Fortin et al., 2012) and BluePyOpt 1.9.126 (Van Geit et al., 2016) python libraries. Score functions were implemented using Blue Brain Project's Electrophys Feature Extract Library 3.2.4 (eFEL)^8^ (Van Geit et al., 2016) and Allen Institutes's IPFX^9^. The CPU based neuron simulations were run using NEURON 7.6.7 .mod files and the NEURON python interface^10^. the software versions and requirements are added as Supplementary Materials. For GPU based neuron simulations we used NeuroGPU 1.0^11^ (Ben-Shalom et al., 2022) and CoreNeuron 1.0^12^ (Kumbhar et al., 2019). For the installation of CoreNeuron we used the Intel PGI compiler, version 20.11-0 and we used Cori's cray-python version 3.7.3.2 to avoid compilation issues with anaconda python used in other experiments.

## 3. Results

### 3.1. Experimental Design

**Figure 3:**
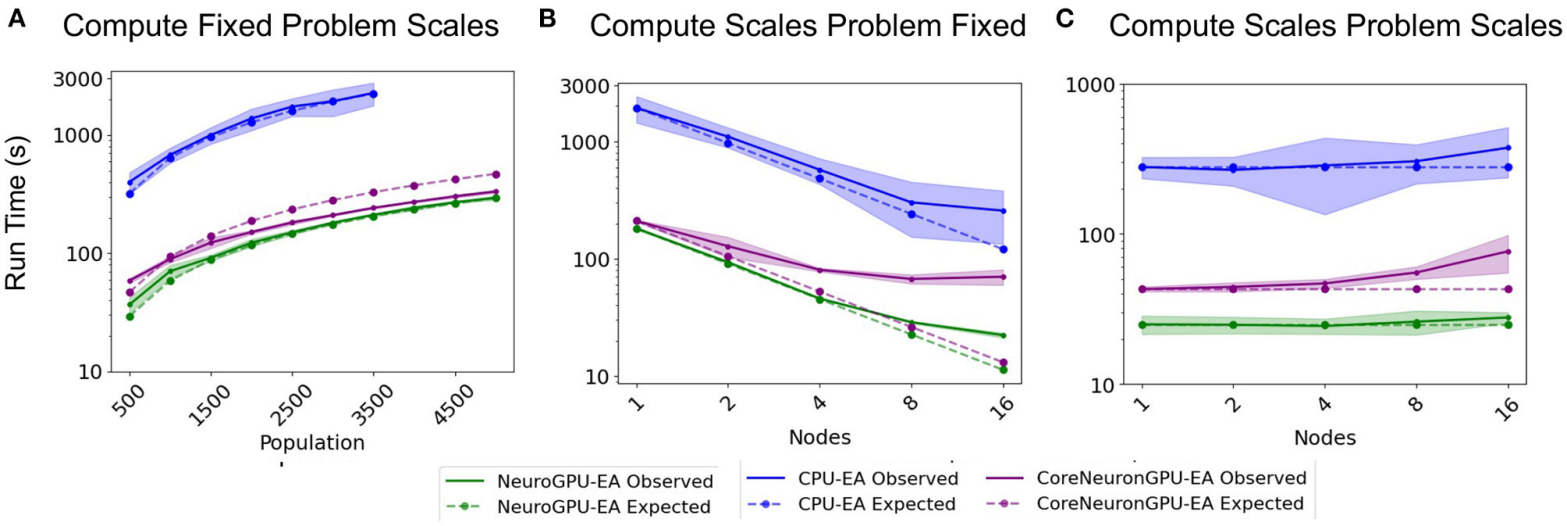
Simulation-evaluation scaling CPU vs. GPU: Experiments measuring the time it takes to run one simulation-evaluation step using *NeuroGPU-EA, CoreNeuron-EA*, and *CPU-EA*. **(A)** One compute node and population size increases, as in Table 1. **(B)** Increases compute nodes and population size is constant, as in Table 2. **(C)** Increasing compute nodes and population size, as in Table 3.

**Figure 4:**
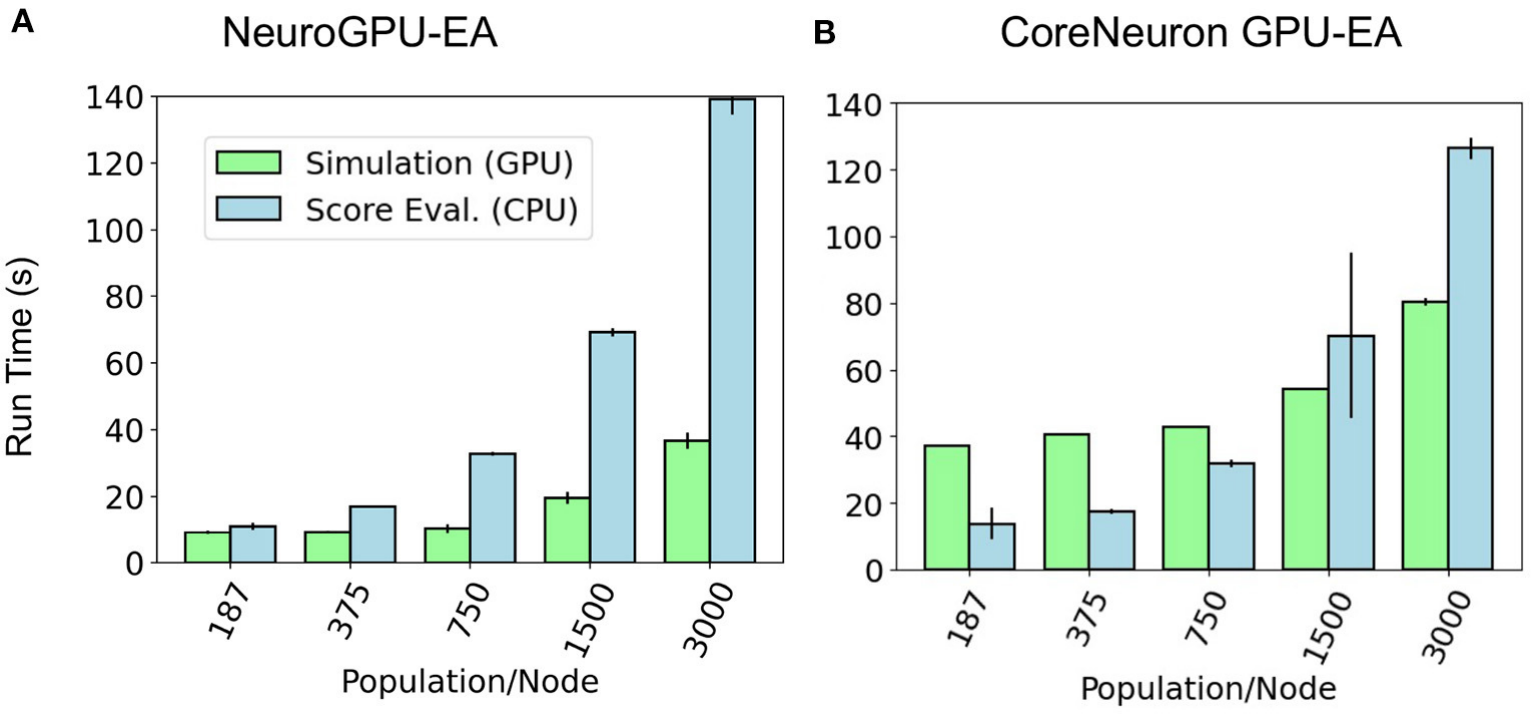
GPU bottleneck shifts to CPU as population per node increases: at large population sizes the CPU operation for score functions is the bottleneck—denoted by relatively taller bars for CPU Eval. At smaller population sizes in **(B)** the GPU simulation is the bottleneck for *CoreNeuron-EA*—denoted by relatively taller bars for simulation. CPU and GPU time are balanced at small population sizes for *NeuroGPU-EA* in **(A)**.

The primary metric of EA performance was the wall time needed to complete one simulation-evaluation step. The three main experimental contexts are *NeuroGPU-EA, CoreNeuron-EA*, and *CPU-EA*. The version of *NeuroGPU-EA* that uses CoreNeuron is termed *CoreNeuron-EA*. We refer to both *NeuroGPU-EA* and *CoreNeuron-EA* as *GPU-EA* to represent GPU based evolutionary algorithms. *CPU-EA* experiments are run on CPU only nodes with 64 single-threaded cores. Unlike simulators used in *GPU-EA, CPU-EA* using NEURON offers an adaptive timestep option, with command h.cvode_active(1), which allows the simulator to perform fewer integration solves when there are fewer spikes. *CPU-EA* uses the h.cvode_active(1) setting for applicable stimuli as this setting accelerates NEURON simulation time. As demonstrated in Supplementary Figure 1, NEURON with adaptive timestep had a notably faster average simulation time than standard NEURON settings. For benchmarking experiments, 50 trials were run using an initial population with the same seed. Supplementary Figure 2 shows that running *NeuroGPU-EA* trials with multiple seeds resulted in a slight speedup for the 500 and 1,000 populations, but also resulted in more deviation between recorded times for these population sizes. For all experiments, the first generation of every optimization was discarded so the time spent loading the morphology of model neurons was not included. Morphology loading time was not included for *GPU-EA* because NeuroGPU begins with a mapping of the model in the GPU, while CoreNeuron had an initial cost of 0.35 s per model to load the morphology^13^. Further benchmarking of how different topologies, models, and morphologies affect simulation run time can be found in Figures 3, 4 of previous work (Ben-Shalom et al., 2013). The *GPU-EA* model transfer to CPU was not intentionally benchmarked either, as the NeuroGPU model only exists on the GPU. However, logs from CoreNeuron trials indicate an average cost of 3 ms for moving a single model to the GPU. Furthermore, CoreNeuron outputs indicated that a single model used 560 kB of memory. CPU experiments that were not ran for enough trials are not represented. We report the mean and standard deviation of the run time. We provide run time lower bounds as ideal scaling measures, in accordance with Hoefler and Belli (2015). To confirm these benchmarks are practicable, we include the optimized model responses and statistics at key generations for EA with population size 1,000 in Supplementary Materials.

### 3.2. Benchmark 1

The “Compute Fixed Problem Scales” benchmark measures the computational capacity of the algorithm with a fixed resource allocation. The problem scales with increases in the population size, N, at increments of 500 until the population size reaches 5,000. “Compute Fixed” means using 64 CPUs on one node for the *CPU-EA* algorithm and using 80 CPUs together with 8 GPUs for the *GPU-EA* algorithm. The results from this benchmark experiment are shown in Figure 3A and Table 1. Across all population sizes, *CPU-EA* took 10x the amount of time it took *NeuroGPU-EA* to complete a simulation-evaluation step and 7x the amount of time it took *CoreNeuron-EA* to complete a simulation-evaluation step. The comparative performance of *CoreNeuron-EA* and *CPU-EA* aligns with previous benchmarking studies showing CoreNeuron's 2-7x decrease of NEURON execution time (Kumbhar et al., 2019). Between *GPU-EA* experiments in Figure 3A, *NeuroGPU-EA* had approximately 20% speed-up when compared against *CoreNeuron-EA*. Supplementary Figure 3A shows that both feature extraction libraries had similar scaling performance, with Allen IPFX extractor running slightly faster in general, exhibiting a speed up of about 10%. Supplementary Figure 4A shows this experimental design applied to *NeuroGPU-EA* using Oak Ridge National Lab's (ORNL) Summit^14^ computing cluster. Experiments ran on Cori showed a speed-up of around 20% when compared to those ran on Summit. These experiments characterize the rate in which run time of simulation-evaluation loop grows as the population size scales up. There are several possible scaling bounds such as logarithmic *O*(*log*(*N*)), linear *O*(*N*), polynomial *O*(*N*^*k*^), and exponential *O*(*k*^*N*^) where *k* is a constant and *N* is the population size. Our expectation is that the run time of the algorithm should increase directly proportional to the increase of the population size. This would be a linear relationship or *O*(*N*). Figure 3A and Supplementary Figures 3A, 4A all confirm a close alignment between expected scaling performance and actualized scaling performance. To further investigate the factors that drive an increase in run time in the application, additional experiments analyzing single node performance were required.

**Table 1:** Compute fixed and problem scales: Stimuli and score functions are fixed 8 and 20, respectively.

**Population**	**NeuroGPU-EA run time (s)**	**CPU-EA run time (s)**	**CoreNeuronGPU-EA run time (s)**
500	36.8 ± 5.71	401 ± 82.4	58.7 ± 2.72
1,000	70.6 ± 8.83	679 ± 98.6	89.2 ± 5.25
1,500	91.6 ± 5.52	1,000 ± 159	123 ± 12.8
2,000	123 ± 8.88	1,380 ± 285	151 ± 53
2,500	150 ± 6.48	1,740 ± 296	182 ± 6.92
3,000	182 ± 3.98	1,930 ± 490	210 ± 3.58
3,500	212 ± 3.56	2,270 ± 494	242 ± 3.72
4,000	242 ± 10.4	-	272 ± 3.78
4,500	270 ± 4.87	-	304 ± 7.77
5,000	295 ± 11.8	-	333 ± 9.52

*Each experiment uses one node. CPU node has 64 cores. GPU nodes have 80 CPU cores and 8 GPUs. ± values indicate 1 standard deviation*.

Further motivation for scaling population size on a single node is that this analysis can identify bottlenecks that occur at different problem sizes. Figure 4 shows a set of experiments ranging from 187 to 3,000 neurons models per node using *GPU-EA* and *CoreNeuron-EA*. These experiments measured the run time for simulating (GPU) and evaluating (CPU). In this figure, both GPU computations and CPU computations are potential bottlenecks. Starting at around 375 individuals per node, up to twice as much time is spent running simulations on the GPU than evaluating them on the CPU. The proportion of run time on the CPU to run time on the GPU increases with the amount of population per node. At 3,000 individuals per node, the CPU evaluation takes twice as long as the GPU evaluation time. For both *CoreNeuron-EA* and *NeuroGPU-EA*, when the population size is larger than 1,000, the CPU is the bottleneck. Thus, predicting the simulate-evaluate run time as population per node increases becomes increasingly dependent on CPU run time.

### 3.3. Benchmark 2

The second benchmark, “Compute Scales Problem Fixed”, determines the *strong scaling* of the application. Keeping the problem size constant and increasing the number of allocated CPU/GPU resources quantifies the potential for parallelism to accelerate the simulation-evaluation step. In this experimental design, specified by Table 2, the population size, N, is fixed at 3,000, while the number of allocated nodes scales up by a factor of 2. The outcomes for scaling N exponentially are represented in Figure 3B. The expectation is that run time decreases exponentially by a factor of 2, corresponding to the compute scaling rate. For all GPU-based algorithms, after 4 nodes, or 2 nodes in the case of *CoreNeuron-EA*, run time acceleration per node starts to decrease and no longer match expected scaling. This demonstrates a limit to which parallelism in *GPU-EA* can efficiently leverage available resources. As shown in Figures 4A,B and Table 2, at 187 individuals per node the time to complete evaluation is around 14 and 16 s for 375 individuals. This demonstrates Amdahl's law (Amdahl, 1967) which states that the overall improvement gained by parallelized code is limited to the fraction of time that code is in use. The benefit of parallelizing across the population and electrophysiological score functions is limited by the time the slowest score function takes to complete. Similarly, for simulation, both *GPU-EA* and *CPU-EA* show marginal decrease in run time after population size begins decreasing below 750 individuals per node. These results support the analysis shown in Figure 3B, as the benefit of using more than 4 nodes to simulate and evaluate 3,000 neurons is limited by the speed of the software modules deployed in the respective tasks. The equivalent experiments, shown in Supplementary Figures 3B, 4B, using IPFX electrophysiological score functions and different computing architecture demonstrate the same limitations in using more than 4 nodes to simulate 3,000 neuron models. The next benchmark illustrates how scaling the problem size enables efficient utilization of larger resource allocations.

**Table 2:** Compute scales and problem fixed: Stimuli and electrophysiological score functions are fixed to 8 and 20, respectively.

	**CPU node**	**GPU node**		**Run time (s)**		
**Nodes**	**Total CPUs**	**Total CPUs**	**Total GPUs**	**CPU-EA**	**NeuroGPU-EA**	**CoreNeuronGPU-EA**
1	64	80	8	1930 ± 490	182 ± 3.98	210 ± 3.58
2	128	160	16	1100 ± 212	93.6 ± 1.52	128 ± 24.9
4	256	320	32	573 ± 141	45.9 ± 0.586	80.6 ± 2.64
8	512	640	64	302 ± 149	28.9 ± 0.526	67.4 ± 5.96
16	1,024	1280	128	257 ± 123	22.4 ± 1.05	70.4 ± 10.5

*Population size is fixed to 3,000. each*.

### 3.4. Benchmark 3

The third benchmark, “Compute Scales Problem Scales”, determines the *weak scaling* of the application. In this experimental design, specified by Table 3, the initial trial sets a scaling factor 250 population (*N* = 250) per node. The subsequent trials increase the number of CPU/GPU nodes and population size proportionally. The expectation is that run time remains constant. These experiments demonstrate how multi-node parallelism can accommodate the scaling of population size in the evolutionary algorithm. As demonstrated in Figure 3C, scaling at 250 individuals per node allows the run time of algorithm to remain approximately constant for up to 10 nodes. We chose to scale at 250 individuals per node because in this allocation the time spent on the GPU and CPU are nearly balanced for *GPU-EA*. Furthermore, this choice of scaling factor resulted in a higher average GPU utilization, at around 70%, as demonstrated in Supplementary Figure 6. This figure demonstrates the proportion of time spent running computations on the GPU and CPU compared to the total run time. With a scaling constant of 250 individuals, at more than 10 nodes the run time starts to marginally increase with each trial. In *CPU-EA*, the increase in run time is marginal. Supplementary Figure 3C demonstrates that eFEL score functions and Allen IPFX provide both match the expected constant scaling and the performance is nearly identical. The IPFX library is a few seconds faster than eFEL. Further experiments, shown in Supplementary Figures 3C, 4C, demonstrate that overhead is incurred when *NeuroGPU-EA* is run on larger allocations of GPU nodes (64–128 Nodes) using the Summit computing cluster. In the Section 4, further consideration is taken toward the explaining implications of successful large-scale optimization runs and the software/hardware that powers such runs.

**Table 3:** Compute scales and problem scales: see Table 2 for other details.

	**CPU node**	**GPU node**			**Run time (s)**		
**Nodes**	**Total CPUs**	**Total CPUs**	**Total GPUs**	**Population**	**CPU-EA**	**NeuroGPU-EA**	**CoreNeuronGPU-EA**
1	64	80	8	250	279 ± 44.9	25.0 ± 3.49	42.9 ± 1.53
2	128	160	16	500	267 ± 58	24.8 ± 3.13	44.3 ± 3.08
4	256	320	32	1,000	285 ± 151	24.4 ± 2.85	46.8 ± 3.24
8	512	640	64	2,000	305 ± 88.4	26.0 ± 4.75	55.3 ± 5.29
16	1,024	1,280	128	4,000	374 ± 137	27.7 ± 2.24	76.5 ± 21.4

### 3.5. Scaling Stimuli and Electrophysiological Score Functions

**Figure 5:**
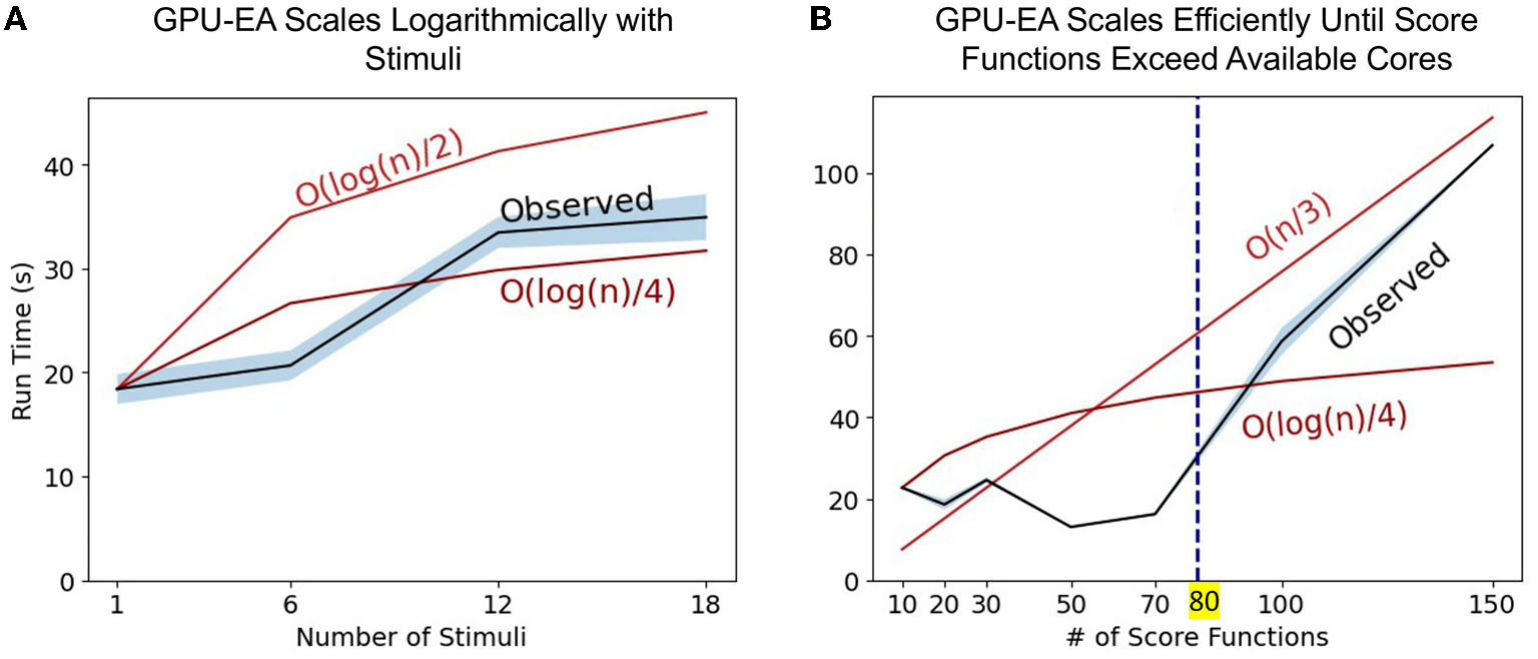
Scaling stimuli and electrophysiological scoring functions: Panel **(A)** represents the observed run time as the number of stimuli used in the algorithm increases. We provide two lines for scaling reference $O\left(\frac{log\left(n\right)}{2}\right)$ and $O\left(\frac{log\left(n\right)}{4}\right)$. Panel **(B)** represents the observed run time as the number of score functions used in the algorithm increases. We provide two lines for reference, $O\left(\frac{n}{3}\right)$ and $O\left(\frac{log\left(n\right)}{4}\right)$.

The set of experiments above only changes the problem size using population size, N. To further explore the axes of scaling *GPU-EA* problem space, we ran scaling experiments on *GPU-EA* with *NeuroGPU-EA* where electrophysiological score functions are set to 20, population size is set to 500 but the number of stimuli used in EA increases from 1 to 18. This experiment is shown in Figure 5A. In this figure, we use big O notation to denote worst case scaling of running time. The $O\left(\frac{log\left(n\right)}{2}\right)$ and $O\left(\frac{log\left(n\right)}{4}\right)$ lines show the starting run time scaled by the log transform of the expected increase in run time. This figure shows that *GPU-EA* scales logarithmically with the number of stimuli used. Furthermore, we ran an experiment on *GPU-EA* where the number of stimuli is fixed to 8, population size is fixed to 500 but the number of electrophysiological score functions used in EA increases from 1 to 180. This experiment is shown in Figure 5B. In this figure, there is constant scaling for up to 80 score functions. Once the number of electrophysiological score functions exceeds 80 they can no longer run entirely in parallel and the algorithm begins to scale at a constant linear rate—$O\left(\frac{n}{3}\right)$. These results in scaling different dimensions of the EA problem size further demonstrates how computational resources can be leveraged using parallelism and concurrency to achieve efficient scaling in our *GPU-EA* algorithmic design.

### 3.6. Benchmark Model Fit

**Figure 6:**
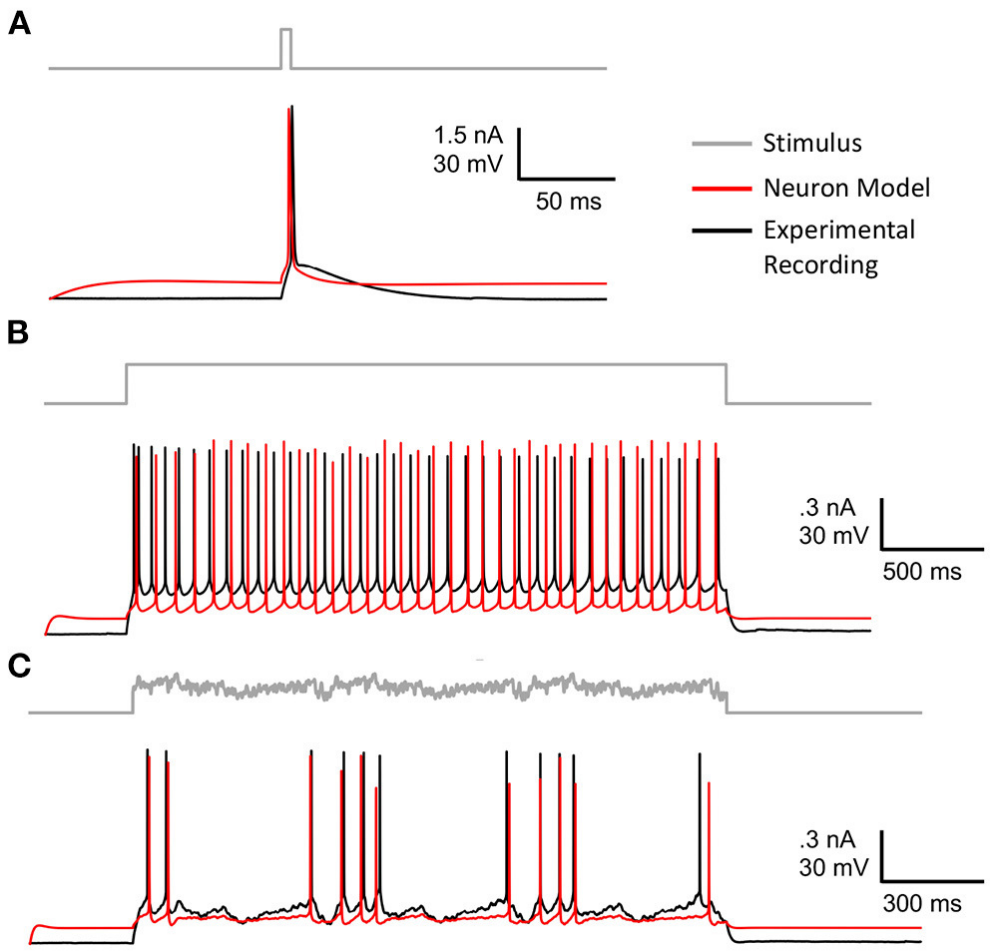
Best fitted model after 50 generations of EA using 8 stimuli and 20 score functions (red) plotted against experimental data (black). **(A)** Long square stimulus. **(B)** Short square stimulus. **(C)** Noisy stimulus. The remaining stimuli and scores are shown in Supplementary Figure 5 and Supplementary Table 1, respectively.

Figure 6 shows the model neuron, specified in Section 2, that was fit using GPU-GA. Congruent with benchmarks 1, 2, and 3, the EA used to fit this model was constrained to use 5,000 population size 20 score functions and 8 stimuli. Models are fitted against publicly available experimental data and stimuli from the Allen Cell Types Database (Gouwens et al., 2019) specimen 488683425. The experimentally recorded cell, plotted in black in Figure 6, is a layer 5 thick tufted pyramidal visual cortex cell. The best model, obtained according to the procedure in Section 2, is plotted in red. Figure 6 shows that the fitted model neuron demonstrates a similar firing rate and spike onsets that are well-aligned with those of experimental data. While the simulated model waveforms closely align with experimental data, the voltage base and after hyperpolarization depth (AHP) vary from those produced by the experimental neuron. As shown in Supplementary Figures 5A,E, the voltage base is indicative of a limitation of the passive dynamics of the optimized model, such as g_pas and e_pas. These dynamics could be alleviated through the use of more appropriate passive score functions. The generalized response of the model to stimuli that were not used in the optimization is also demonstrated in Supplementary Figures 5F–H. These results show the quality of model that can be achieved with the simple EA design and stimuli used in the benchmarks, however there are many aspects of EA optimization that can be tuned to achieve an improved neuron model. This is why a general understanding of optimization quality from different EA configurations is important. For instance, the beneficial impact of scaling EA population size is exemplified in the next section.

### 3.7. Effect of Scaling Up EA Population

**Figure 7:**
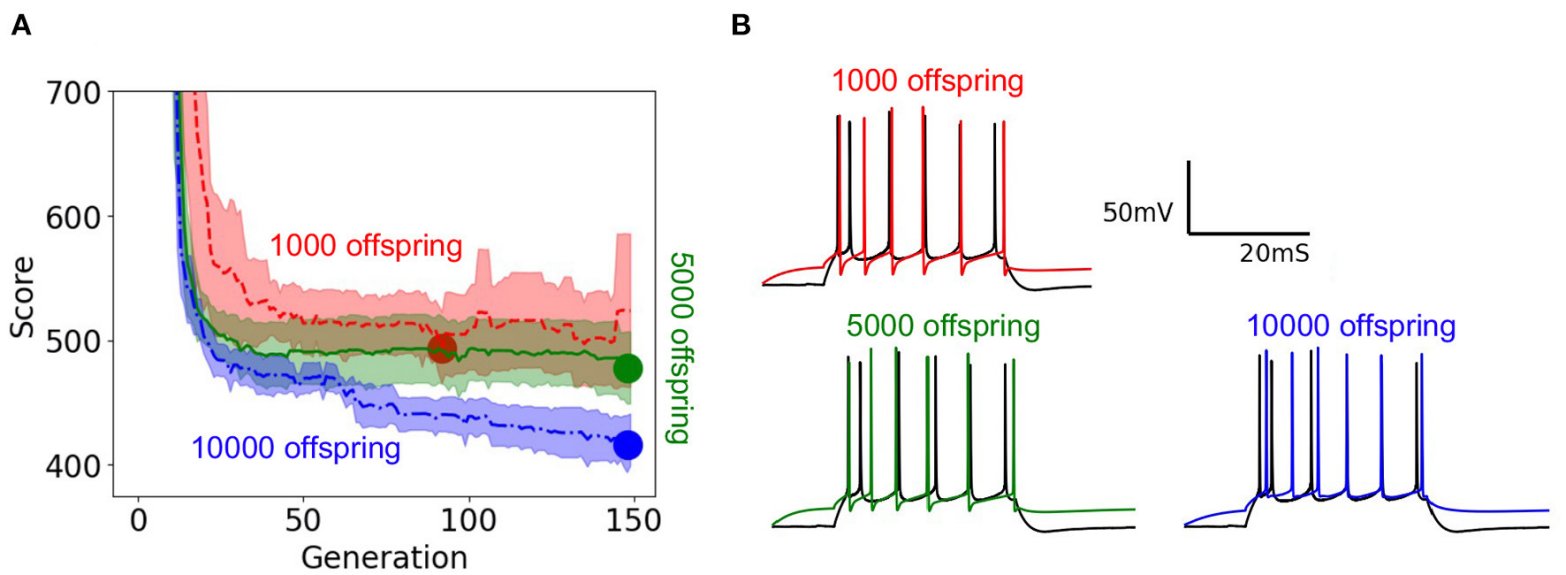
EA score and model fit both improve with larger population size: **(A)** Objective function optimization trajectory in EA with varying population sizes. Scores start around 2,500 but the y-axis is constrained to clearly show results. Lower scores indicate a closer fit to experimental data. The minima of the objective function are denoted by large circles and the lower the minima the more the best simulated response resembles the experimentally recorded waveform. Confidence intervals are computed using 10 random initializations. Panel **(B)** illustrates the neuron model responses corresponding to varying population sizes.

To demonstrate the practical impact of scaling the evolutionary algorithm, we set up an experiment on the layer 5 thick tufted pyramidal neuron from the Blue Brain Project (Ramaswamy et al., 2015) described in Section 2. The purpose of this experiment is to demonstrate the benefit of increasing population size on the resulting optimized model. In this experiment, we ran 150 generations of the evolutionary algorithm for three different trials with population sizes 1,000, 5,000, 10,000, respectively. Unlike the EA in Section 3.6 which utilized the 8 stimuli from benchmarks 1–3, this version of EA utilized 8 different long square currents, as shown in Figure 1A, and 8 score functions per stimulus. Six of the score functions are represented in Figure 1C and the other two are minor variations of the rendered score functions. For each population size, we ran 10 trials using 10 different random seeds for EA. As a Monte Carlo method, the trajectory of EA is stochastic, thus using random seeds ensures the score trajectories follow a reproducible trend. Notably, the run time per generation increased as EA progressed toward more optimal parameter sets. By breaking down the cumulative run time associated with running multiple generations of EA, Supplementary Figure 8 shows that as EA progresses, the electrophysiological features take more time to compute on average. Regarding the optimization procedure, Figure 7A demonstrates the mean and 95% confidence interval of the score of the objective function for the evolutionary algorithm at each generation. The 10,000 individual EA achieves a better fit to experimental data, resulting in the lowest achieved value for the objective function. The value for objective function represents a penalty against simulated neurons where the electrophysiological features of voltage traces differ from those of an experimentally recorded target waveform. The lower scores achieved by the 10,000 individual EA indicate this configuration finds comparatively more optimal models for generations 70–150. Compared to 1,000 individual EA, the 5,000 individual EA achieved a lower mean score over 10 random seeds, but this difference was not statistically significant. Furthermore, alignment of the experimentally recorded neuron membrane potential and that of the best simulated neuron model substantiates the impact of improved optimization of the objective function due to larger population size in the EA. In Figure 7B, as the population sizes increase, models show improvement in the depth and timing of the after hyperpolarization (AHP). The AHP depth is the maximum level of depolarization after the action potential has peaked and re-polarized to resting potential. In the 10,000 individual optimization, the AHP depth is not greater than that of the experimentally recorded target waveform. The duration of the hyperpolarization is also more similar to the target waveform for the 10,000 individual optimization than the smaller population size EAs. Figure 7B qualitatively demonstrates that population sizes that allow EA to explore more potential parameter sets construct a model that better characterizes the experimental data (Ben-Shalom et al., 2012). Figure 7A quantitatively supports this claim by showing that EA with 10,000 individuals finds the most optimal solution when compared with smaller population EAs.

## 4. Discussion

The most central comparison drawn in this paper is between CPU and GPU based simulation-evaluation loops. GPU based simulation is markedly faster and scales better than CPU based simulation. These results suggest that *CPU-EA* may be a reasonable choice for fitting simple electrophysiological neuron models, but that researchers should use caution in more computationally complex optimization problems that require scaling. For these complex problems, leveraging parallel code design and GPU neuron simulation can reduce EA optimization time from weeks or days to hours. Based off the CPU experiments in the Compute Fixed Problem Scales section, using a single desktop computer without a GPU would limit researchers to a population size of 1,000 or smaller. Based off the single node GPU example, the addition of a single GPU allows for a researcher to complete 50 iterations of a population size of 3,000 in several days. A conservative estimate is that using *GPU-EA* with a workstation that has 8 GPUs and over 40 cores enables a researcher to complete 50 iterations of an EA with 3,000 individuals in a day. Using the maximum amount of resources available, 128 nodes on Summit, we show in Supplementary Figure 4C that we can simulate and evaluate a population size of 32,000 in 35 s. This allocation makes it feasible to reach 50 generations of 32,000 individuals within the course of a few hours. While these estimates demonstrate the potential scale and advantages of *GPU-EA*, there are instances where *CPU-EA* may be more optimal. In Ben-Shalom et al. (2022), we show that simulating models with less compartments and channels are not substantially accelerated by GPUs. Furthermore, for EA optimizations with only one stimulus and a nominal number of score functions will not benefit from the score function level parallelism and stimuli level parallelism discussed in the Section 2. Finally, for researchers attempting to simulate many different models, NEURON provides the highest level of compatibility with most available models in ModelDB (Hines et al., 2004), though CoreNeuron is expanding its compatibility with NEURON.

Our choice of an appropriate scaling factor was critical in achieving large scale simulation and evaluation. We used the experiment shown in Figure 4A in the *Compute Fixed Problem Scales* section to determine a reasonable scaling constant of 250 neuron models per node. We chose this constant as the simulation and evaluation time were approximately balanced so neither simulation or evaluation would dominate run time. Relative to other configurations, 250 models per node led to the most efficient GPU utilization, at around 60%. 60% is the highest achieved GPU utilization using *GPU-EA* because the GPU must be idle while the evaluation step is finishing. Once all the models are scored and a new population evolves, the GPU resumes activity at 100% utilization. This is shown in the plot of GPU utilization over time in Supplementary Figure 7. Future work could involve implementations that achieve higher GPU utilization through different implementations, such as parallel EAs (PEAs) that evolve multiple sub-populations simultaneously (Cantú-Paz, 2001; Du et al., 2013). It may be necessary to modify GPU simulation modules in order to adapt *GPU-EA* to enable PEAs, or simultaneous EAs with different seeds. Based on Figure 4A, 250 neuron models per node was a conservative choice of a scaling constant, as the CPU did not start to become a bottleneck until 750 population per node and 1,500 population per node in Figure 4B. This conservative choice ensured we would be able to efficiently scale the problem size with number of computing nodes, which we aim to demonstrate for the purpose of benchmarking. In practice, researchers might choose larger scaling constants.

While *GPU-EA*'s ability to leverage parallelized kernel computation for fast simulation is one advantage. Another advantage is that *GPU-EA*, using NeuroGPU, also adds concurrency to the algorithm described in Section 2. Concurrency, defined as the capacity to run separate tasks at the same time (Roscoe, 1998), is different than the achieved levels of multi-node and single node parallelism. This algorithm is concurrent when simulation of the remaining batches of stimuli begin as soon as the first set of stimuli finish. The GPU does not remain idle as the CPU finishes evaluating the first batch of simulations. Thus, while the CPU is evaluating the quality of the simulations, the GPU begins the next batch of simulations. This is shown in Figure 2C as the CPU and GPU are running at the same time. The result of concurrency in *GPU-EA* is shown in Figure 5A where the algorithm scales logarithmically with the number of stimuli used in the algorithm. Logarithmic scaling is enabled by NeuroGPU's capacity to run stimuli in parallel across GPUs as well the algorithmic design to simulate a second set of neuronal models while the previous set of stimuli is being evaluated. This logarithmic scaling enables the objective function of evolutionary algorithm to incorporate multiple stimuli. Consequently, models that are fit using multiple stimuli will generalize better to new unseen stimuli. While state of the art fitting procedures like (Gouwens et al., 2018) are currently designed to use a single stimulus in the optimization algorithm, the addition of simulate-evaluate concurrency can enhance these methods using more stimuli with minimal cost in run time. A challenge with incorporating more stimuli in the objective function is that simulators that don't permit concurrent execution will need to simulate and evaluate sequentially.

In the section *Compute Fixed Problem Scales* and Figure 4, we showed that at too large of a population size, the score functions will bottleneck simulation-evaluation run time. We also found that this bottleneck in the evaluation step can be mitigated or worsened by the number of electrophysiological scoring functions used. Figure 5B showed constant scaling for up to 80 score functions. This happened because there were 80 cores available on a single Cori node. Once the number of score functions exceeds the number of cores available to a node they can no longer be run entirely in parallel and the algorithm begins to scale at a constant linear rate—O(n/3). Thus, if researchers intend to use multiple score functions for multi-objective optimization, as in Druckmann et al. (2007), we recommend they consider using fewer score functions than cores available in *GPU-EA*. Even in the case where there are fewer score functions than cores, Supplementary Figure 8 shows that as EA progresses, the evaluation step takes more time to complete. A potential cause for increased evaluation time is that in later generations there are more spiking neuron models to be evaluated and eFEL score functions take longer on traces with more spikes. These results demonstrate a distinct advantage in simulating larger populations of neurons on GPU nodes as there are many opportunities to implement parallelism and concurrency. However, CPU processing capacity for scoring electrophysiological features hinders the efficient scaling of the *GPU-EA* algorithm. A potential strategy to alleviate this bottleneck could involve loading simulated traces into the score function library before mapping the score functions to be evaluated in parallel. Currently, traces are loaded separately for each score function. Another potential mitigation could be using GPU feature extraction. To the best of the author's knowledge, there are no available GPU based software toolkits for scoring features of simulated spike trains based on a target train. There are several GPU-based applications that are used for real time analysis of Electroencephalography (EEG) waveform data, Magnetoencephalography (MEG) data, and Multi-electrode Arrays (MEA) signals (Tadel et al., 2011; Guzman et al., 2014; Sahoo et al., 2016), but none that exist for evaluating simulated neuron firing traces. Software capable of scoring electrophysiological traces on a GPU would considerably enhance the performance of *GPU-EA* configurations where score functions are the bottleneck. The prospective advantage of GPU accelerated electrophysiological feature extraction presents an opportunity for researchers. Because the Blue Brain Project's eFEL (Van Geit et al., 2016) score function library is developed in C++, it has the potential to be adapted to the GPU through tools like OpenACC's GPU directives.

A critical consideration in attempting to generalize benchmarks, whether between simulation software, HPC platforms, or algorithms, is that factors from the hardware and software environment to the number of spiking neurons in a population can have a substantial impact on the run time of the application. In Supplementary Figure 8, the time to evaluate score functions increased as the EA algorithm produced more spiking neurons. The stochasticity in the optimization in EA is not a desirable property for benchmarking as it can be difficult to tell if scoring is taking longer to complete because it is slower or because an instantiation of EA is producing offspring that spike more. We mitigate this issue by benchmarking one initial population in 3.2, 3.3, and 3.4. Another example of variability in performance occurred in our comparison between Cori and Summit. Initial experiments demonstrated a much more dramatic speedup, but after upgrading to use GCC 8.3.0 (version used on CORI), the performance on Summit improved considerably. Kumbhar et al. (2019) shows a notable increase in performance of CoreNeuron using the Intel C/C++ compiler instead of GCC/G++. Moving from benchmarking stand-alone software modules to applications means there are more dependencies that can be affected by the installation environment. With this consideration we provide a simplified code example^15^ to run one simulation evaluation loop without HPC or EA. We also provide the entire code suite^16^, which we hope to further extend to be a platform capable of benchmarking of more tools in computational neuroscience.

In future work, we aim to apply this benchmarking framework across several other software modules of interest. A simple extension of this work would be to run experiments comparing Allen IPFX and BluePyopt's eFEL to the widely adopted python electrophysiological toolkit Elephant (Denker et al., 2018). While Elephant has fewer statistic-based features, it offers correlative measures between spike trains. Also, Elephant has a parallel extension which can further advantage HPC resources. Another simple extension of this work could involve benchmarking the biophysical neuron simulator LFPy (Lindén et al., 2014) or Arbor (Abi Akar et al., 2019). A more complex extension of this work would involve benchmarking the same simulate-evaluate loop, but as it applies to spiking neural networks (SNN) instead of the evolutionary algorithm. There are several well-documented and widely adopted SNN packages such as Brian (Goodman and Brette, 2009), NEST (Gewaltig and Diesmann, 2007), and SpiNNaker (Furber et al., 2014) that would be appropriate to benchmark using this experimental design. Finally, we are interested in generalizing this benchmarking experimental design to a wider range of single neuron and network optimization tasks. Any algorithm that involves simulation or feed-forward stage and then an evaluation/feedback/learning stage is amenable to the analysis conducted in this paper. This generalizability extends to many methods commonly used in machine learning and optimization.

## 5. Conclusion

This work demonstrates the potential of efficiently parallelized simulation and evaluation software for electrophysiological modeling. Specifically, applications that leverage GPU utilization demonstrate the capacity to run larger fitting optimizations. In turn, these optimizations can result in a larger search of the parameter space, and consequently, a more accurate model. As the processor count continues to increase on hyper-threaded and multi-core chips, computational methods that leverage parallelism can continue to leverage new innovations in high performance computing to generate more detailed and accurate neuronal models. While this progression is beneficial, it is ever relevant to apply established benchmarks such as weak scaling and strong scaling for neuroscientists to get the most value out of new computing resources.

## Data Availability Statement

The datasets presented in this study can be found in online repositories. The names of the repository/repositories and accession number(s) can be found below: https://portal.nersc.gov/cfs/m2043/benchmarking_ea.tar.gz.

## Author Contributions

JB, KB, and RB-S helped the conceptualize experiments. AL and KK designed the software to run, process, and visualize experiments. AL wrote the original draft. RB-S helped with visualization. RB-S and KB funded the project and provided supercomputing hours. All authors have read and agreed to the published version of the manuscript.

## Funding

This research was supported by the MIND Institute and Neurology Department at the University of California Davis and the Action Potential grant from The FamiliesSCN2A Foundation.

## Conflict of Interest

The authors declare that the research was conducted in the absence of any commercial or financial relationships that could be construed as a potential conflict of interest.

## Publisher's Note

All claims expressed in this article are solely those of the authors and do not necessarily represent those of their affiliated organizations, or those of the publisher, the editors and the reviewers. Any product that may be evaluated in this article, or claim that may be made by its manufacturer, is not guaranteed or endorsed by the publisher.

## Footnotes

^1^ https://docs.nersc.gov/systems/cori/

^2^ https://github.com/BlueBrain/BluePyOpt

^3^ https://deap.readthedocs.io/en/master/api/algo.html#deap.algorithms.eaMuPlusLambda

^4^ https://efel.readthedocs.io/en/latest/

^5^ https://bbp.epfl.ch/nmc-portal/downloads.html

^6^ https://ipyparallel.readthedocs.io/en/latest/

^7^ https://docs-dev.nersc.gov/cgpu/

^8^ https://github.com/BlueBrain/eFEL

^9^ https://github.com/AllenInstitute/ipfx

^10^ https://neuron.yale.edu/neuron/

^11^ https://github.com/roybens/NeuroGPU

^12^ https://github.com/BlueBrain/CoreNeuron

^13^ https://github.com/BlueBrain/CoreNeuron/issues/642

^14^ https://www.olcf.ornl.gov/olcf-resources/compute-systems/summit/

^15^ https://github.com/xanderladd/benchmarking_examples

^16^ https://github.com/xanderladd/EA_benchmarking
