# Supplementary material for: Scaling and Benchmarking an Evolutionary Algorithm for Constructing Biophysical Neuronal Models
Source: Front Neuroinform. 2022 Jun 17;16:882552. doi: 10.3389/fninf.2022.882552 (PMC9248031; doi:10.3389/fninf.2022.882552)
Supplement: Supplementary file 1 [file Data_Sheet_1.pdf]

5.1 Supp. Figs

Parameter	Lower Bound	Upper Bound	Base Value
g_pas_all	0.000003	0.00300	0.000300
e_pas_all	-150.0	-50.0	-75.0
gNaTa_tbar_NaTa_t_axonal	0.313797	31.37968	3.137968
gK_Tstbar_K_Tst_axonal	0.008926	2.89259	0.089259
gNap_Et2bar_Nap_Et2_axonal	0.000683	0.06827	0.006827
gK_Pstbar_K_Pst_axonal	0.097354	9.73538	0.973538
gSKv3_1bar_SKv3_1_axonal	0.102194	10.21945	1.021945
gCa_LVAstbar_Ca_LVAst_axonal	0.000875	0.08752	0.008752
gCa_HVAbar_Ca_HVA_axonal	0.000099	0.00990	0.000990
gSKv3_1bar_SKv3_1_somatic	0.030347	3.03472	0.303472
gCa_HVAbar_Ca_HVA_somatic	0.000099	0.00994	0.000994
gNaTs2_tbar_NaTs2_t_somatic	0.098395	9.83955	0.983955
gCa_LVAstbar_Ca_LVAst_somatic	0.000033	0.03330	0.000333

Table S1: Free parameters optimized during EA. Names are formatted by parameter, mechanism, and section. All units are in S/cm^2.

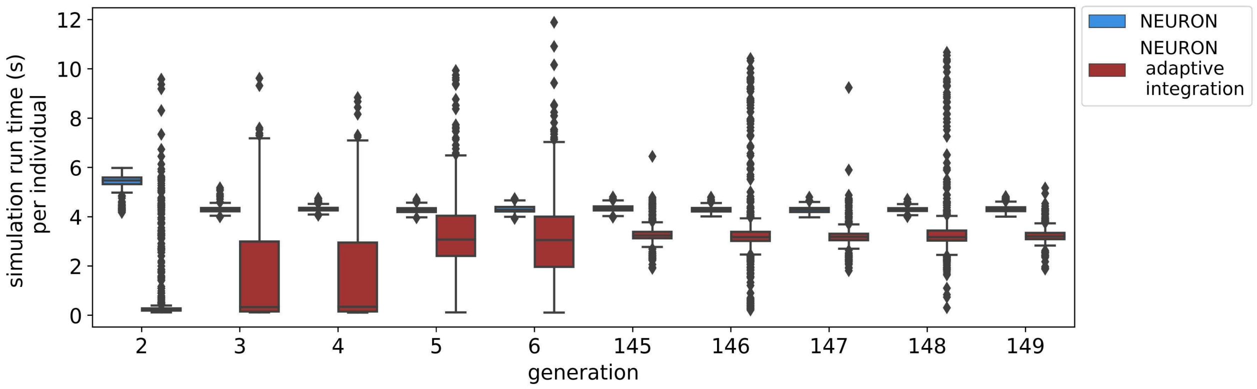

Figure S1: Distribution of simulation run time per neuron model using NEURON with and without adaptive integration. Run time per model was computed using one long square stimuli. The fixed timestep NEURON model uses a timestep of 2 microseconds. Neuron model parameter sets were loaded from previous optimizations in Section 3.7. Fixed timestep NEURON for generations 2-6 had an average per model run time of 4.52 +/- .48 seconds compared to adaptive timestep which had an average per model run time of 2.01 +/- 1.97 seconds. Fixed timestep NEURON for generations 145-149 had an average per model run time of 4.32 +/- .13 seconds compared to adaptive timestep which had an average per model run time of 3.33 +/- .99 seconds. +/- values are standard deviation.

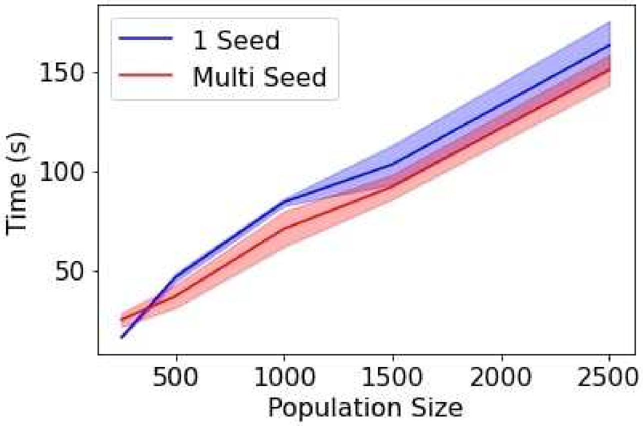

Figure S2: First 5 experiments of Compute Fixed Problem Scales benchmark described in Section 3.2 for sets of trials that used different starting seeds and the same starting seed respectively. The single seed benchmark runs several seconds slower than the multi-seed benchmark at population sizes 500 and 1000, otherwise there is no significant difference between multi-seed and single seed benchmarks.

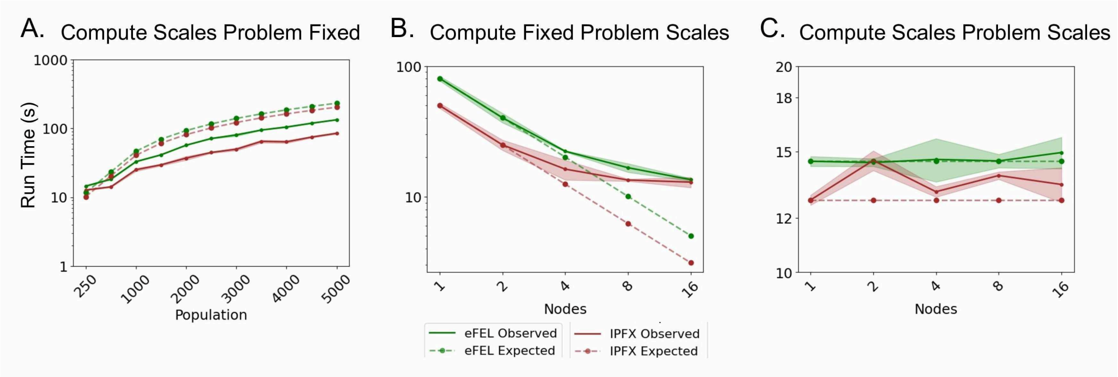

Figure S3: **S3A, S3B, S3C:** Compares EA simulate-evaluate run time between Blue Brain Project's eFEL score function library and the Allen Institute's IPFX score function library. All experiments in this figure use the NeuroGPU simulator. Unlike Figure 3, 8 score functions were used instead of 20. **Note**: In some figures the y axis is logarithmically scaled to show exponential scaling and in some figures it is not.

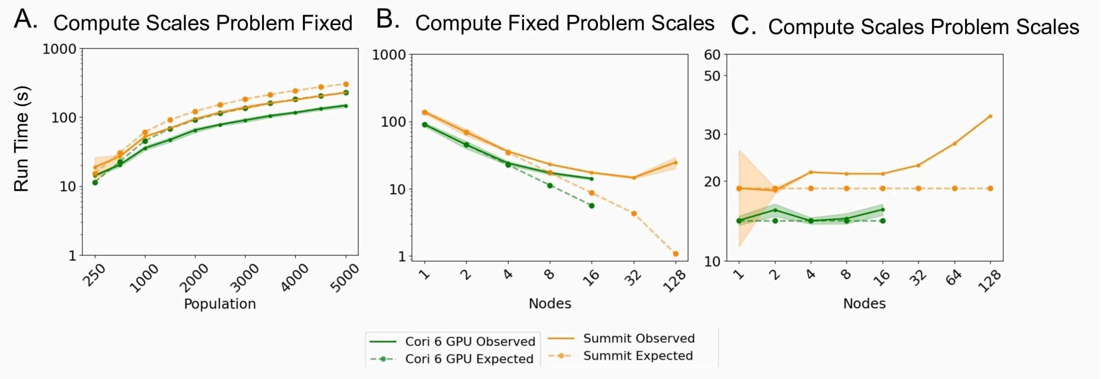

Figure S4: **S4A, S4B, S4C:** Compares EA simulate-evaluate run time between NERSC's Cori with 6 GPUs and Oakrdidge National Lab's (ORNL) Summit cluster. All experiments in this figure use the NeuroGPU simulator and the experimental layout described in Table 1 an Table 2 in the methods section

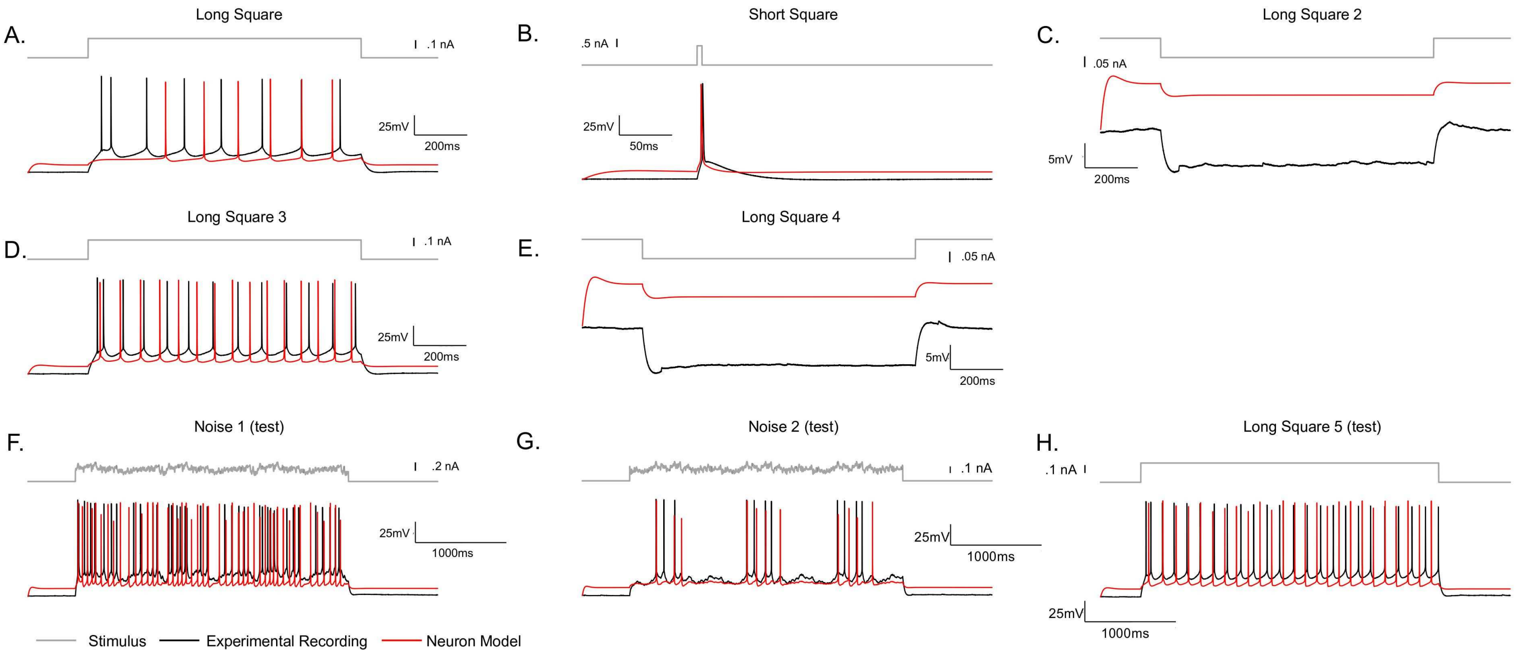

Figure S5: **A-E:** Remaining stimuli used in the optimization in Section 3.6. Black traces are the experimentally recorded target data. Red traces are simulated by the neuron model. Gray traces represent stimuli. **F-H** the neuron model's generalized response on stimuli that were not used in the optimization.

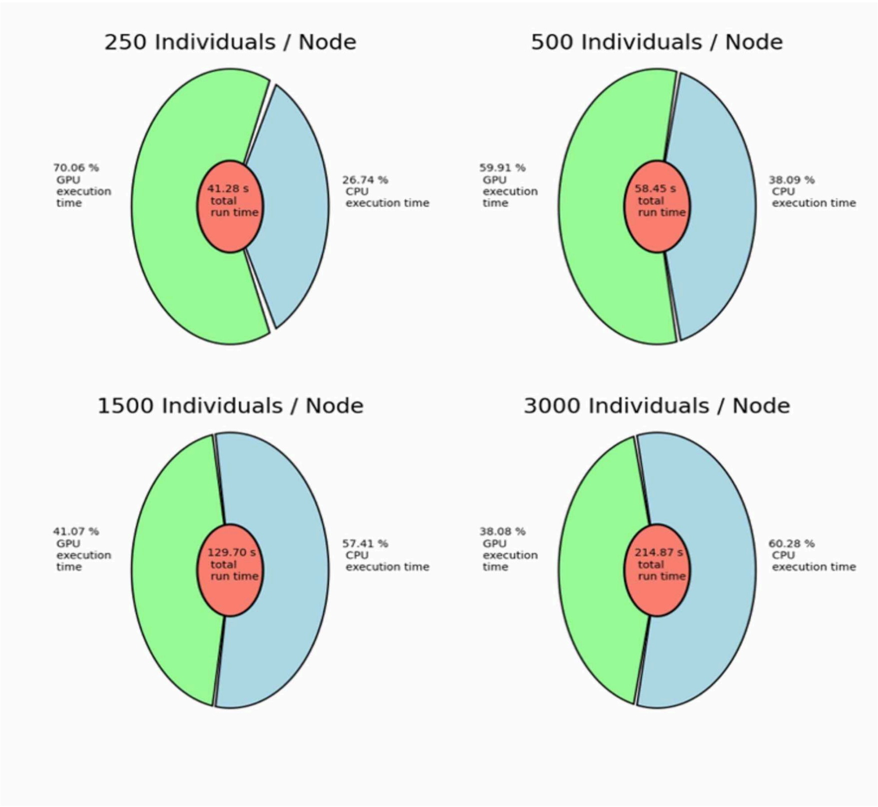

Figure S6: CPU and GPU time as a fraction of the total wall time for various population per node configurations. The larger the population size is, the more time is spent on the CPU calculating the objective function.

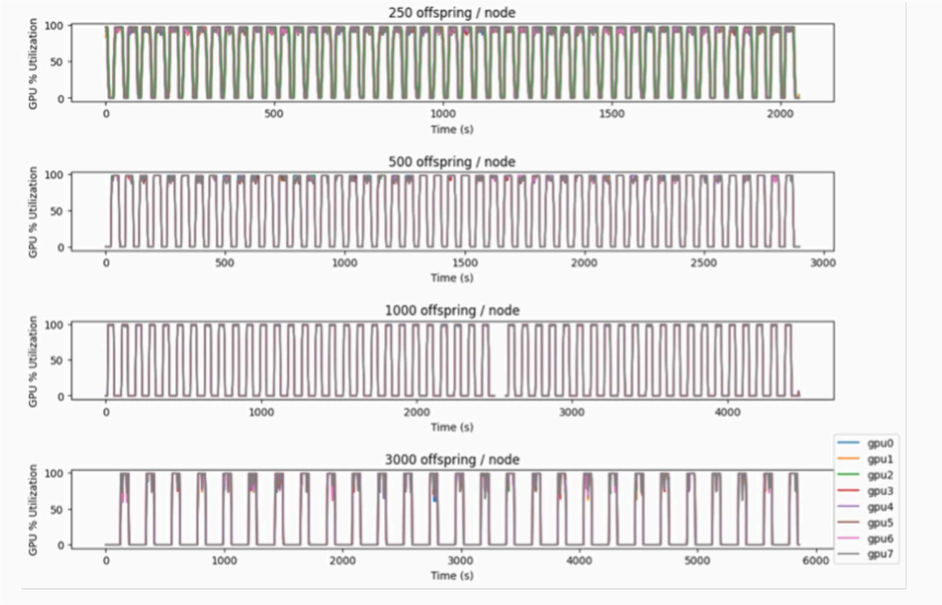

Figure S7: GPU utilization over time for different population sizes. When the GPU is active it is at 100% utilization. The inactive troughs happen while the GPU is being blocked by the score functions phase of EA in algorithm 2

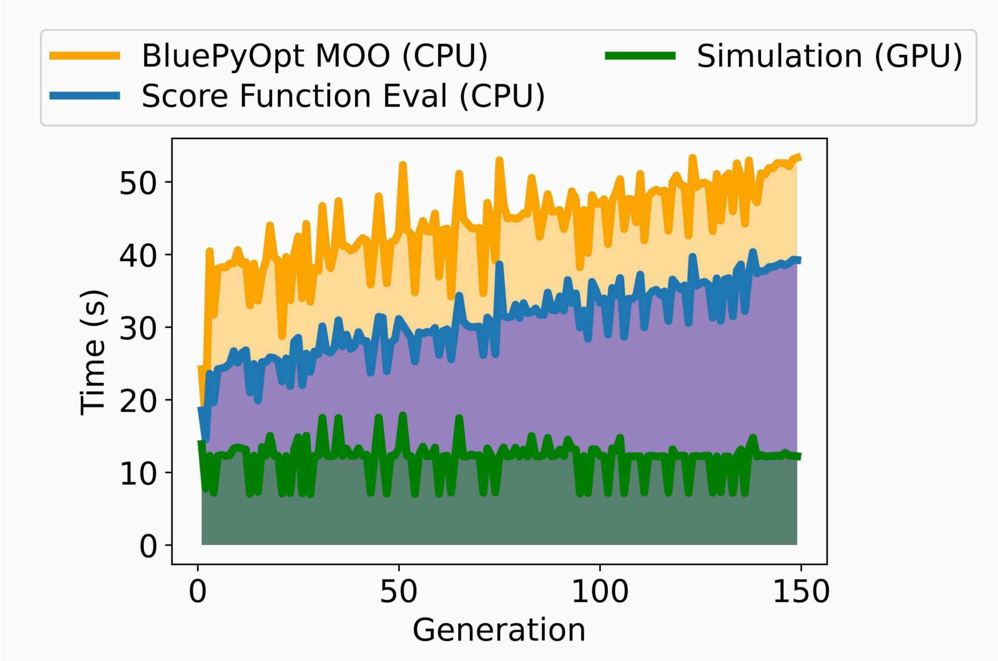

Figure S8: Breakdown of run time for 5000 individual EA optimization trials in Figure 7 by time spent in MOO, simulation, and score function evaluation for *NeuroGPU-EA*. Time spent in MOO and GPU remains generally constant but score function evaluation time increases in later generations. Times at each generation were averaged over several random seeds. The max evaluation time was 61 seconds, the mean was 18 seconds and the standard deviation was 12 seconds
